# Multiple genes contribute to anhydrobiosis (tolerance to extreme desiccation) in the nematode *Panagrolaimus superbus*

**DOI:** 10.1590/1678-4685-GMB-2017-0030

**Published:** 2017-11-06

**Authors:** Cláudia Carolina Silva Evangelista, Giovanna Vieira Guidelli, Gustavo Borges, Thais Fenz Araujo, Tiago Alves Jorge de Souza, Ubiraci Pereira da Costa Neves, Alan Tunnacliffe, Tiago Campos Pereira

**Affiliations:** 1 Departamento de Biologia, Faculdade de Filosofia, Ciências e Letras de Ribeirão Preto, Universidade de São Paulo (USP), Ribeirão Preto, SP, Brazil; 2 Programa de Pós-Graduação em Genética, Faculdade de Medicina de Ribeirão Preto, Universidade de São Paulo (USP), Ribeirão Preto, SP, Brazil; 3 Departamento de Física, Faculdade de Filosofia, Ciências e Letras de Ribeirão Preto, Universidade de São Paulo (USP), Ribeirão Preto, SP, Brazil; 4 Deptartment of Chemical Engineering and Biotechnology, University of Cambridge, Cambridge, UK

**Keywords:** peroxiredoxin, kinase, RNAi, proteostasis

## Abstract

The molecular basis of anhydrobiosis, the state of suspended animation entered by some species during extreme desiccation, is still poorly understood despite a number of transcriptome and proteome studies. We therefore conducted functional screening by RNA interference (RNAi) for genes involved in anhydrobiosis in the holo-anhydrobiotic nematode *Panagrolaimus superbus*. A new method of survival analysis, based on staining, and proof-of-principle RNAi experiments confirmed a role for genes involved in oxidative stress tolerance, while a novel medium-scale RNAi workflow identified a further 40 anhydrobiosis-associated genes, including several involved in proteostasis, DNA repair and signal transduction pathways. This suggests that multiple genes contribute to anhydrobiosis in *P. superbus*.

## Introduction

A few species of bacteria, yeasts, plants and small invertebrates are capable of surviving extreme desiccation through a unique and outstanding strategy: anhydrobiosis. When facing severe drought, these species begin to dehydrate and, instead of dying, they accumulate intrinsically disordered proteins (such as LEA and TDPs; Boothby *et al.*, 2017) and non-reducing disaccharides (such as trehalose and sucrose), which promote the vitrification of the internal cellular environment. This process results in a bioglass – an amorphous organic scaffold that completely arrests metabolism and preserves internal contents (Crowe *et al.*, 1998). It is also proposed that such disaccharides act as `water-replacement molecules’, directly interacting with proteins and membranes and helping to maintain their native structures (Sakurai *et al.*, 2008). However, it is possible that other factors also contribute to the protection of the organism. In this “dry state” (anhydrobiosis itself), the organism is tolerant to several other physical stresses such as extremes of temperature and pressure, ultraviolet light and radiation (Tunnacliffe and Lapinski, 2003; Watanabe *et al.*, 2006a,b).

In rotifers, anhydrobiosis arrests the “biological clock”, meaning that they do not age when in suspended animation. Therefore, the average lifespan is unaltered by desiccation, regardless of the time spent in the dry state (Ricci *et al.*, 1987). However, once a dried rotifer is rehydrated, the same animal needs an interval of at least 24 h before being subjected to desiccation again to be able to survive (Schramm and Becker, 1987). Notably, variations in the life histories of different anhydrobiotic species may occur (Ricci and Caprioli, 1998).

Anhydrobiotic organisms are exposed to extreme water stress, which causes deleterious effects in the cell, including oxidative damage (Leprince *et al.*, 1994; França *et al*., 2007). Oxidative stress refers to a biological condition in which there is an imbalance in the concentrations of oxidant species and antioxidants (Sies, 2000). Organisms have developed adaptive mechanisms for cellular detoxification, including systems that repair or prevent damage caused by oxidants (Michiels *et al*., 1994). Among such defense systems in the anhydrobiotic nematode *Panagrolaimus superbus* are: protein DJ-1 (Culleton *et al.*, 2015), glutathione peroxidases (GP114) and peroxiredoxin (PER).

Although anhydrobiosis in animals was first described more than three centuries ago (Van Leeuwenhoek, 1702), its molecular basis is poorly understood. Transcriptome and proteome analyses in tardigrades (*Milnesium tardigradum*, *Richtersius coronifer* and *Hypsibius dujardini*), bdelloid rotifers (*Adineta ricciae*), nematodes (*Aphelenchus avenae*, *Ditylenchus africanus*, *Plectus murrayi* and *Panagrolaimus superbus*), insects (*Polypedilum vanderplanki*), algae and plants (*Pyropia orbicularis, Myrothamnus flabellifolia* and *Boea hygrometrica*) identified several genes and proteins that were up- or down-regulated by water loss (Adhikari *et al.*, 2009; Haegeman *et al.*, 2009; Mali *et al.*, 2010; Schokraie *et al.*, 2010, 2012; Boschetti *et al.*, 2011; Tyson *et al.*, 2012; Yamaguchi *et al.*, 2012, Wang *et al.*, 2014; López-Cristoffanini *et al.*, 2015; Ma *et al.*, 2015; Zhu *et al.*, 2015; Ryabova *et al.*, 2017). Such studies are, by their nature, correlative, and do not provide evidence for a functional role of the genes or proteins concerned. In the model nematode, *Caenorhabditis elegans*, whose dauer larvae alone are desiccation tolerant, a large number of mutants are available that can be used to study anhydrobiosis in this species (Erkut *et al.*, 2011, 2013). However, in other nematodes, the lack of such mutants has prompted researchers to use RNA interference (RNAi) techniques instead (Reardon *et al.*, 2010), an approach which is also possible in *C. elegans* (Gal *et al.*, 2004; Erkut *et al.*, 2013).

Identification of anhydrobiosis-related genes is a central requirement in the development of anhydrobiotic engineering, which aims to confer desiccation tolerance on dehydration-sensitive biological samples (cells, tissues, organs) (Chen *et al.*, 2009; García De Castro *et al.*, 2000; Li *et al.*, 2012). Successful anhydrobiotic engineering would have multiple applications in agriculture (*e.g*., by rendering plants tolerant to drought) and medicine (*e.g*., preservation in the dry state of organs for transplant).

In this study we examined anhydrobiosis in *P. superbus*, a free-living nematode nearly 1 mm long that feeds on bacteria and was first described by Fuchs (1930). Members of the genus *Panagrolaimus* inhabit diverse niches, from the Antarctic, volcanic islands, temperate and semi-arid soils to terrestrial mosses (Shannon *et al.*, 2005; McGill *et al.*, 2015). *P. superbus* and *C. elegans* belong to the same order (Rhabditida) and are anatomically similar. However, the former is dioecious while the latter is typically hermaphroditic and has a faster populational growth rate. We have focused on *P. superbus*, rather than *C. elegans*, because the former nematode is: (i) holo-anhydrobiotic (Jönsson, 2005) i.e., able to enter anhydrobiosis at any life stage, (ii) robustly desiccation tolerant (Shannon *et al.*, 2005) and (iii) does not demand extra/special laboratory procedures to obtain specific larval stages.

We first developed a new method for rapid and accurate assessment of survival to desiccation that could be used in a scalable screening procedure. To test this method and also to gain further information on the functional roles of glutathione peroxidase (a protein previously shown to be involved in anhydrobiosis; Reardon *et al.*, 2010) and peroxiredoxin (a biochemically related protein), we performed RNA interference by feeding on populations of *P. superbus*. We then developed a new medium-scale RNAi screening protocol to screen a panel of 97 target genes previously shown to be regulated during extreme desiccation in other anhydrobiotic species and found that knockdown of 40 of these genes adversely affects desiccation tolerance in *P. superbus*.

## Materials and Methods

### Nematode maintenance

*Panagrolaimus superbus* (strain DF5050) used in this study was first isolated from Surtsey Island (Iceland) by Björn Sohlenius (Sohlenius, 1988; Shannon *et al.*, 2005) and was maintained in incubators at 21 °C, in the dark, on NGM (Nematode Growth Medium) agar plates and fed with a layer of *Escherichia coli* (strain OP50).

### Evaluation of staining for the determination of survival percentages

*P. superbus* worms were collected from maintenance plates (NGM agar covered with a layer of OP50) by rinsing with 5 mL of M9 buffer, transferred to 50 mL test tubes and left for 10 min to precipitate. Then, the supernatant was discarded and worms were transferred to new 50 mL tubes containing 10 mL of M9 buffer. This washing procedure was done three times to reduce the amount of OP50 bacteria. Worms were then separated into two 0.2 mL vials. One sample was heated at 70 °C for 10 min in a thermocycler with subsequent decanting for 10 min. Heating at this temperature is lethal to worms, providing a positive control for staining. The second sample was kept at rest for 20 min at room temperature.

Supernatant was then removed and 180 μL trypan blue or erythrosin B (both 0.4% w/v in M9 buffer) were added. Tubes were left at room temperature for four hours without agitation. After this period, supernatant was removed and 150 μL of M9 buffer were added; the total volume was transferred to 1.5 mL tubes, to which 850 μL of M9 was added. After gentle agitation, tubes were left without agitation for 10 min for worm decantation. This washing procedure (disposing supernatant and adding 950 μL of M9 buffer) was repeated three times to remove the dye. Subsequently, worms were placed on plastic plates.

For each treatment, three plates were produced: i) a sample of live worms, ii) a sample of dead worms, and iii) a mixture of live and dead worms. These plates were analyzed via light microscopy and images were captured using the program ScopePhoto to verify whether stained worms (bluish by trypan blue, pink by erythrosin B) were active, thus revealing any false positives. Similarly, a total and permanent absence of movements in unstained (live worms), representing false negatives, was also considered. The whole procedure was also performed for *C. elegans* worms. Staining for only one hour was also tested. Three biological replicates were performed (N > 100 worms per group, per replicate).

### RNAi by feeding - PER and GP114

Partial cDNAs corresponding to a glutathione peroxidase (designated GP114, GenBank Accession Number GR881191) and peroxiredoxin (designated PER, GenBank Accession Number GR881190) in the L4440 vector (ampicillin resistance) were propagated in *E. coli* HT115 (Reardon *et al.*, 2010). These feeding strains, designated dsGP114 and dsPER, were grown in 50 mL tubes of liquid LB ampicillin (50 μg/mL) under agitation (210 rpm, 37 °C) overnight. Subsequently, tubes were centrifuged for 10 min at 3,500 x *g*. Pellets were resuspended with 600 μL of liquid LB ampicillin and inverted on petri dishes containing NGM agar and IPTG (1 mM). Plates were left for two days at room temperature to induce double-stranded RNA (dsRNA) expression by the bacteria.

Worms were then collected from OP50 and washed with M9, as previously described. Subsequently, a small amount of worms was transferred to either plates containing bacteria expressing dsRNA against GP114 or PER. They were left for nearly 15 days to ensure silencing of the entire population. HT115 bacteria containing a GFP gene cloned in the L4440 vector (L4440::GFP, referred to as “GFP”) was used as a negative control. Populations fed with L4440::GP114 bacteria are referred to as “dsGP114”, “GP114 knockdown” or “GP114-silenced”; similarly for PER, *mutatis mutandis*. The terms “GP114” and “PER” were used to refer to the corresponding genes/cDNAs. Three biological replicates were performed (N = 200 worms for each treatment, for each replicate).

### Desiccation challenge

Worms were submitted to desiccation challenge according to Shannon *et al.* (2005). Briefly, silenced worms were immobilized on 0.45 μm Supor filter membranes (Sigma Aldrich) by vacuum filtration with a Sartorius funnel, placed in 1.5 mL test tubes and then subjected to the following conditions: 98% relative humidity (RH) for 24 h over a saturated solution of copper sulphate (unless otherwise stated); 10% RH for 24 h over dry silica gel and pre-hydration in 100% RH for 24 h in distilled water vapour. Rehydration was achieved by adding 1.5 mL of M9 buffer to the samples. Survival percentage was measured by staining with erythrosin B. Three biological replicates were performed (N > 100 worms per group, per replicate).

### Assessing the roles of PER and GP114 as antioxidants

PER- and GP114-silenced worms (in M9 buffer) were subjected to oxidative stress by adding hydrogen peroxide (H_2_O_2_, Synth) to the following final concentrations: 0 μM (zero), 1 μM, 10 μM, 100 μM, 1 mM, 10 mM, 20 mM and 40 mM. The final volume in all tubes was 100 μL. These values were selected according to previous studies on *C. elegans* (Larsen, 1993).

Samples were then homogenized by mild agitation and incubated at 20 °C for 24 h. After this period, the supernatant was removed and 1 mL of erythrosin B (0.4% w/v) was added and left for four hours. Worms were then washed three times with M9 buffer and survival percentages were determined (number of unstained worms/total number of worms). Three biological replicates were performed (N = 200 per group, per replicate).

### Screening for anhydrobiosis-related genes in *P. superbus*

#### Selection of targets

A total of 97 potential targets were considered for the screening experiments. The first group comprised 33 kinase-related cDNAs, obtained from a mixed population of *P. superbus* and cloned in the pDNR-Lib vector (Clontech), kindly provided by Dr. Trevor Tyson (Van Andel Institute, USA). They correspond to all genes whose “target codes” end with a “K”, in Table 1. These targets were selected because signaling processes are likely to be very important for entry into anhydrobiosis. The second group (all other genes in Table 1) comprised 64 genes shown to be up-regulated during anhydrobiosis in other animal species (Adhikari *et al.*, 2009; Haegeman *et al.*, 2009; Mali *et al.*, 2010; Schokraie *et al.*, 2010, 2012; Cornette *et al.*, 2010; Boschetti *et al.*, 2012; Tyson *et al.*, 2012; Yamaguchi *et al.*, 2012). These targets were selected by considering the following aspects: (i) they should be induced in at least one species during anhydrobiosis and (ii) there should be homolog(s) within the *P. superbus* EST library (Tyson *et al.*, 2012).

**Table 1:** Functional identification of anhydrobiosis-related genes via RNAi. All the 40 genes whose knockdown lead to statistically significant decreases in survival percentage ≤10% compared to control group (*i.e.*, worms soaked with RNA duplexes against GFP, normalized as 100% survival) are listed here in gray. Nine genes (marked with asterisks) presented statistically significant decreases lower than 10% (One-Way ANOVA). The remaining 48 targets that did not lead to statistically significant reductions in the initial screenings and are listed in white.

Target Code	Target identity
1K	putative serine threonine-protein kinase (7e-25)
2K	Cyclic AMP-dependent protein kinase
3K	Casein kinase II regulatory subunit
4K	Protein kinase
5K	casein kinase I isoform gamma-1 (9e-19)
6K	C2 domain containing protein (4e-62); CBR-FER-1 protein (2e-46); myoferlin (8e-20)
7K	TKL/LISK/TESK protein kinase (2e-64)
8K	diacylglycerol kinase (5e-83)
9K	PREDICTED: serine/threonine-protein kinase Nek6-like (1e-62)
10K	CK1/WORM6 protein kinase (2e-141)
11K	SH3-domain kinase binding protein
12K	Serine/threonine-protein kinase
13K	testis-specific serine threonine-protein kinase 2 (5e-58)
15K	malonyl-acyl carrier protein (2e-22). ADP-specific phosphofructokinase/glucokinase conserved region family protein (5e-22)
16K	serine threonine protein kinase-related domain containing protein (4e-49)
17K	putative tyrosine-protein kinase kin-31 (6e-83); SH2 motif and tyrosine protein kinase and protein of unknown function DUF595 domain containing protein (5e-81)
18K	gastrulation defective protein 1 (9e-54); protein kinase domain containing protein (6e-50)
19K	adenylate kinase 1 (6e-28)
20K	serine threonine-protein kinase pelle (1e-58); CBR-PIK-1 protein (1e-57)
21K	Er (fms/fps related) protein kinase
22K	Cyclin-dependent kinases regulatory subunit
23K	Guanylate kinase family protein
24K	protein MAK-1, isoform c (7e-11)
26K	Protein kinase domain containing protein
27K	CAMK/CAMKL/MELK protein kinase (2e-27)
28K	serine threonine-protein kinase akt-1 (3e-90)
33K	putative tyrosine-protein kinase kin-31 (1e-12)
34K	protein kinase domain-containing protein (7e-78); casein kinase I isoform gamma-1 (3e-76)
36K	TK/FER protein kinase (5e-61)
37K	tyrosine-protein kinase fer (1e-47)
38K	CK1/TTBKL protein kinase (7e-48)
39K	serine threonine protein kinase-related domain containing protein (6e-53)
40K	PLK/PLK1 protein kinase (5e-51)
si23	Pinin/SDK/memA/ protein conserved region containing protein
si24*	glutamate dehydrogenase (1e-06)
si25	cathepsin L-like cysteine proteinase (4e-150)
si26	A - heat shock protein 70
si27/28	B/C - Heat shock 70 kDa protein
si29	Ras-related protein Rab-1A
si30	Ras-related protein Rab-11B
si31	cuticle collagen protein LON-3 (3e-27)
si32	CBR-RPS-0 protein (40S ribosomal protein AS) (9e-29)
si33	Immunodominant antigen Ov33-3 / Pepsin inhibitor Dit33
si34	Ubiquitin-conjugating enzyme H1
si35*	histone H2B 2 (7e-53)
si36	cytochrome P450 like_TBP (3e-29)
si37	CRE-RPL-9 protein
si38	ribosomal protein L44 (4e-30)
si39	A - euk. Transl. Elong. factor 1A
si40	B - euk. Transl. Elong. factor 1A
si41	Elongation factor 1 beta
si42	DNA repair protein RAD51 homolog 1 (4e-110)
si43	Pv-hsp60
si44	Pv-p23
si45	putative heat shock protein 90 (2e-143)
si46	60S ribosomal protein L4 (1e-147)
si47	40S ribosomal protein S8 (2e-71)
si48	60S ribosomal protein L7a (3e-120)
si76	oxidoreductase, aldo/keto reductase family protein (8e-77)
si77	zinc finger domain containing protein (8e-39) (AN1-like Zinc finger, 7e-37)
si78	channel protein, MIP family (3e-74); aquaporin (3e-69)
si79	autophagy-related protein 2-like protein A (1e-14)
si80	peptidyl-prolyl *cis-trans* isomerase domain containing protein (3e-12); cyclophilin-type peptidyl-prolyl *cis-trans* isomerase-15 (9e-09)
si81*	chaperonin Cpn60 TCP-1 domain containing protein (3e-63)
si82	Derlin-2
si83	DJ-1
si84	Ezrin Radixin Moesin family member (erm-1)
si85	HSP70 cochaperone BAG1
si86	LC3, GABARAP and GATE-16 family member (lgg-1)
si87*	ATP-dependent protease La (1e-75); lon protease homolog, mitochondrial precursor (7e-74)
si88*	isocitrate dehydrogenase, NADP-dependent (7e-102)
si89*	prefoldin subunit 2, PFD-2 (3e-10)
si90	Probable E3 ubiquitin-protein ligase
si91	Proteasome subunit alpha type 4
si92*	CRE-PBS-1 protein (5e-52); proteasome domain containing protein (2e-50)
si93	Protein disulfide isomerase
si94	RIC1 Putative stress responsive protein
si95	Small heat shock proteinalpha crystallin family
si96	tetratricopeptide TPR-1 domain containing protein (3e-43); hsp70-interacting protein, putative (1e-23)
si97	THaumatiN family member
si98	Ubiquitin conjugating enzyme (E2) family member (ubc-3)
si99*	ubiquitin (2e-112)
si100*	ubiquitin-activating enzyme E1 (4e-68)
1	Novel protein (PREDICTED: 1 2-dihydroxy-3-keto-5-methylthiopentene dioxygenase-like)
2	(Lamin Receptor / ribosomal Protein AS)
3	Large subunit ribosomal protein 23
4	Proteasome 26S subunit subunit 4 ATPase
6	Sterol carrier protein
7	Aspartyl protease protein 6
8	Thymidylate synthase
9	ATP synthase subunit family member
10	ADP/ATP translocase
11	Bi-functional glyoxylate cycle protein
15	40S ribosomal protein S12
16	Proteosome subunit alpha
17	Glutathione s-transferase
21	Heat shock protein

#### Production of long double-stranded RNAs (dsRNAs)

dsRNAs were generated for all 33 kinase-related cDNAs. Briefly, 500 ng target cDNAs (cloned in pDNR-Lib vector) were subjected to PCR using primers which correspond to flanking sequences in the vector and include a T7 promoter tail (Table S1). PCR was performed with GoTaq DNA Polymerase (Promega) in 50 μL under the following conditions: 94 °C for 5 min , followed by 33 cycles of 94 °C for 30 s, 53 °C for 30 s, 72 °C for 1 min, and an extension step at 72 °C for 10 min. The resulting amplicons were precipitated with isopropanol, resuspended with ultrapure water and submitted to *in vitro* transcription (Yu *et al.*, 2002) using TranscriptAid T7 High yield transcription kit; ThermoScientific, followed by DNase I treatment, according to the manufacturer’s instructions. dsRNAs were diluted to 1 μg/μL with ultrapure water and Tris HCl (pH 6.8) was added to a final concentration of 5 mM.

dsRNAs were also generated for 14 other genes from *P. superbus* (Table S1, target codes 1-4, 6-11, 15-17 and 21). Initially, their corresponding cDNAs were obtained by RT-PCR (using the same conditions described in section 2.6.5) and cloned into vector pCR2.1 TOPO (Invitrogen). These cloned sequences were then used as templates for a second round of PCR (performed as for pDNR-Lib, described above), but now using T7-gene specific primers (sequences on Table S1; targets 1 to 21). The resulting amplicons could be readily used for *in vitro* transcription (as previously described), yielding dsRNAs.

#### Design of dicer substrates

Dicer substrates were designed for 50 targets (genes whose target codes start with “si”, on Table S2), using the freeware Strand Analysis (Pereira *et al.*, 2007) and extended three nucleotides at each end. These molecules are 27 RNA duplexes, with two nucleotide 3′ overhangs and phosphate groups at the 5′ ends. As a negative control, we designed a dicer substrate against GFP (accession number X83960). Dicer substrates were purchased from Sigma-Aldrich; their sequences are listed in Table S2.

#### RNA interference by soaking with siRNAs/dsRNAs

RNAi was triggered by soaking 200 - 600 worms (per biological replicate per target) for 24 h, in the dark, with long dsRNAs at a final concentration of 0.8 μg/μL (soaking volume: 35 μL) or dicer substrates (siRNAs) at final concentration of 1 μM (soaking volume: 100 μL) and kept in the dark for 24 h without agitation at 21 °C. Three biological replicates were performed.

#### Confirmation of gene silencing by semi-quantitative RT-PCR

We selected a few representative targets to perform semi-quantitative RT-PCR to evaluate gene silencing. We also assessed a representative gene shown not to be involved in anhydrobiosis (si86 – whose silencing did not lead to decrease in survival) to show that a lack of decrease in survival after desiccation is not due to ineffective gene silencing.

Initially, total RNA from worms was extracted using the TRIzol reagent (Invitrogen) according to the manufacturer’s guidelines. RNA samples were quantified by spectrophotometry and subsequently diluted in ultra pure water (RNase free) to yield a final concentration of 1 μg/μL. All RNA samples were pre-treated with DNase I (Fermentas) following a modified version of the manufacturer’s protocol: one unit of enzyme (1 h at 37 °C), followed by addition of another unit of enzyme (1 h at 37 °C). Reverse transcription reactions (RT) were performed using ImProm-II^TM^ kit (Promega) and random primers (500 ng) in a final volume of 20 μL, according to the manufacturer. PCR was then performed using the GoTaq^R^ DNA Polymerase kit (Promega) according to the manufacturer’s instructions. PCR was performed using 2 μL of RT and 25 picomoles of each gene-specific primer (forward or reverse) or β-actin (separate tubes) in a final volume of 50 μL.

All PCR reactions were performed under the following conditions: 94 °C for 5 min, followed by 33 cycles of 94 °C for 30 s, 57 °C for 30 s, 72 °C for 1 min, and a final extension at 72 °C for 10 min. Amplifications curves were analyzed to guarantee that a plateau was not reached under these conditions for the tested genes. PCR products were resolved in 1% agarose gel stained with Sybr Safe (Invitrogen). Band densitometry was done using IMAGE J software. Data normalization was done by dividing the value obtained for the silenced gene by the value found for β-actin for the corresponding sample. For each target, semi-quantitative RT-PCR was performed in technical triplicates, each one consisting of a pool of nearly 600 worms.

Nucleotide sequences of each primer are listed in Table S1. PCR conditions were the same for all genes analyzed, except for target si86: 23 cycles.

#### Lethality assay

In order to determine whether the knockdown alone causes a decrease in viability, worms were soaked for 24 h with RNA duplexes for all 97 targets (or fed with dsPER/dsGP114) and survival percentages were determined by staining. This procedure aims to guarantee that any decrease in survival percentage (compared to control group) is due to the disruption of the process of anhydrobiosis rather than an unrelated lethality. Three biological replicates were performed (N = 200 per group, per replicate).

### Statistical analyses

All experiments were performed in biological triplicates (or quadruplicates) and data are presented as mean values and standard deviations. Statistical analyses were performed using Student’s *t*-test, z-test or one-way ANOVA (with Tukey’s or Dunn’s post-hoc tests) or Mann-Whitney Rank Sum test with SigmaStat software. Statistical differences were considered significant when *p* ≤ 0.05. Only those genes giving a decrease in survival percentage >10% after RNAi/desiccation challenge were considered “anhydrobiosis-related”.

## Results

### Evaluation of staining for the determination of survival percentages

Both tested compounds (erythrosin B and trypan blue) are commonly used for staining cells, and were successfully used to indicate viability in whole worms (Figure S1 A-F). Heat-killed worms were strongly and completely stained, while live animals remained unstained even after four hours of soaking in dye solution. In a very few cases, faint local staining was observed in live nematodes, probably indicating local tissue damage.

After desiccation challenge, all developmental stages (eggs, larvae and adult worms) were stained when dead. No false negatives were observed. However, staining patterns varied: in many cases we observed intense whole-body staining, but in some cases partial staining (of the anterior, middle or posterior body regions) were seen in moving worms (false positives). Light staining occurred in some larvae and adults, without movement, which we considered dead. Staining for four hours or one hour was equally effective. It is possible that desiccation might make membranes leaky without killing worms (generating the few observed false positives). However, we believe that the observed “whole-body staining” reflects a degree of membrane damage that is incompatible with life, and thus, these worms can be considered dead. Additionally, such categorisation was applied to all groups (experimental and control), allowing an unbiased analysis.

### Involvement of a peroxiredoxin gene in *P. superbus* anhydrobiosis

Previously, a glutathione peroxidase gene (GP114) was shown by RNAi to be involved in *P. superbus* anhydrobiosis (Reardon *et al.*, 2010). To test whether the staining method could demonstrate the effect of gene silencing on survival after desiccation, we first fed nematodes bacteria expressing GP114 dsRNA. In addition, we also used RNAi to examine the role of another *P. superbus* antioxidant gene, encoding a peroxiredoxin (PER), in anhydrobiosis.

**Figure 1:**
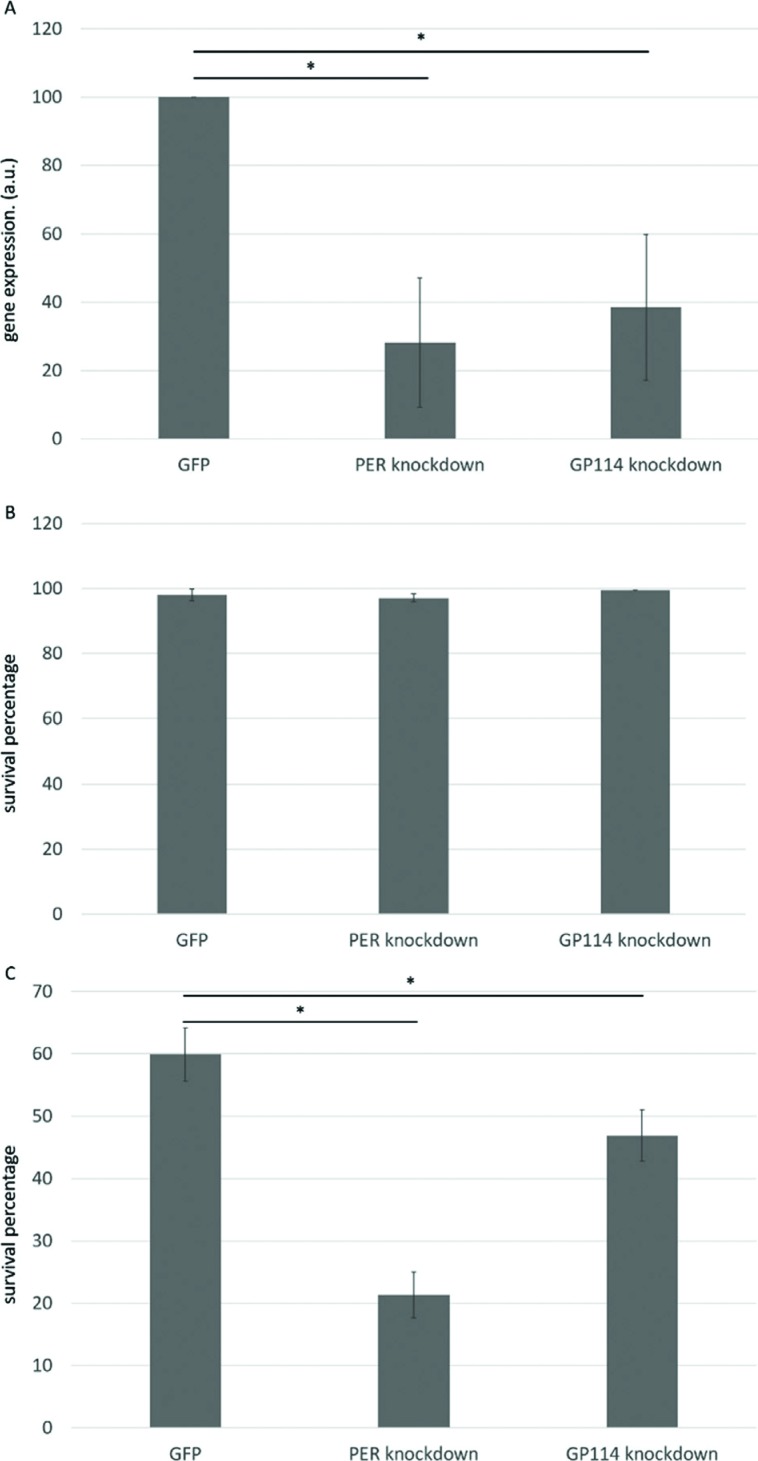
Involvement of peroxiredoxin and glutathione peroxidase in anhydrobiosis in *P. superbus*. A) Molecular analysis by semi-quantitative RT-PCR revealed an average reduction of 71% of PER RNA transcripts and 61% of GP114 in worms subjected to RNAi by feeding compared to the control group corresponding to worms fed with bacteria expressing dsRNA against GFP gene, which is not associated with anhydrobiosis (* *p* ≤ 0.05, one-way ANOVA). B) Viability tests before desiccation. Average survival percentages obtained for different treatments. Groups did not statistically differ and remained above 95% survival. C) Survival tests after extreme desiccation. Silencing PER promoted a 66% reduction in survival percentage when compared to control group; GP114 promoted a 21% reduction (* *p*≤0.05; one-way ANOVA).

Gene silencing was confirmed by semi-quantitative RT-PCR, revealing an average reduction in mRNA levels of 71% for PER and 61% for GP114 transcripts, compared to a control group (Figure 1A). Prior to desiccation, gene silencing had no effect on nematode viability (Figure 1B), demonstrating that PER and GP114 are not essential genes under the tested conditions. After desiccation, all groups displayed increased mortality (Figure 1C), but PER and GP114 groups were significantly more sensitive than the GFP control. In particular, PER knockdown resulted in a 66% decrease in viability compared to the control. As well as confirming a role for glutathione peroxidase, these data also show for the first time the participation of a peroxiredoxin (PER) in nematode anhydrobiosis. An EST-based study on *P. superbus* identified two clusters for peroxiredoxin genes and three for glutathione peroxidase (Tyson *et al.*, 2012). Therefore, it is possible that the lower survival percentage observed when silencing peroxiredoxin, compared to glutathione peroxidase, is due to the lower compensation capacity within the first protein family. These findings are consistent with oxidative stress being a significant component of the various stress vectors experienced by desiccating nematodes, as indicated for other organisms (Haegeman *et al.*, 2009; Cornette *et al.*, 2010). This experiment also validates the staining method for the assessment of survival of desiccation.

### PER and GP114 act as antioxidants

Knockdown of PER and GP114 had little or no effect on the morphology, development, fertility and behaviour of *P. superbus* (data not shown), as anticipated. However, we would expect gene silencing to compromise the ability of nematodes to combat oxidative stress, and we therefore tested this by exposing control and experimental groups to increasing concentrations of hydrogen peroxide for 24 h.

**Figure 2:**
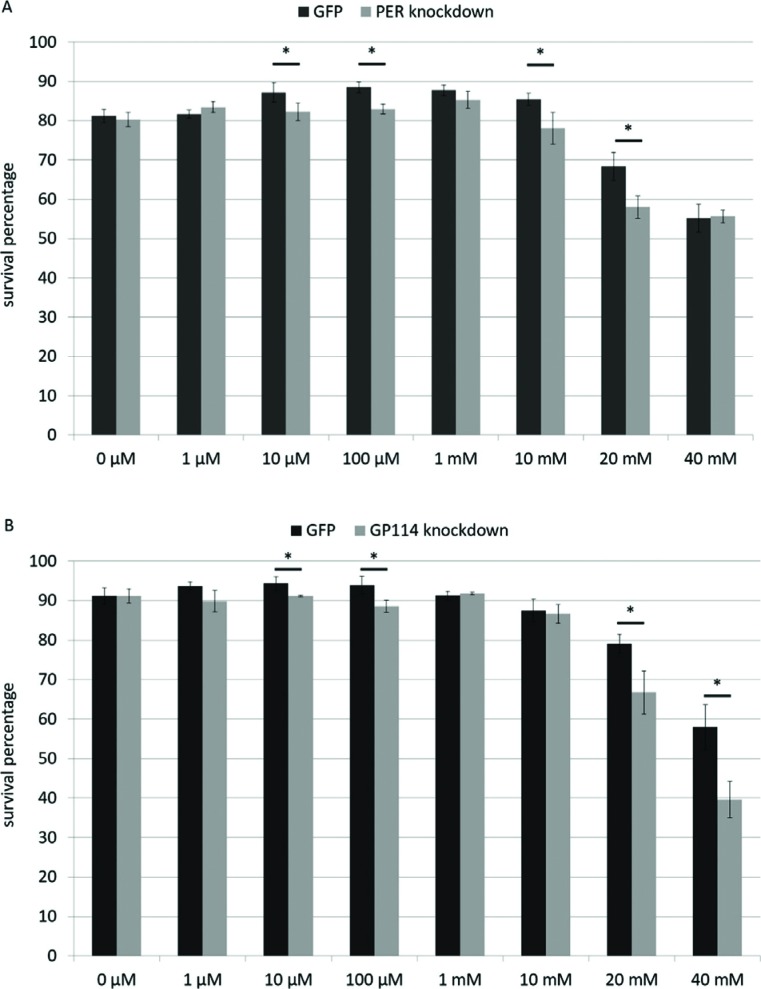
PER and GP114 enzymes combat oxidative stress. Statistically significant decreases in survival percentages were observed in both treatments (glutathione peroxidase- and peroxiredoxin-silenced worms) exposed to hydrogen peroxide. (* p<0.05; t-test between control group (GFP) and corresponding experimental group).

Slightly fluctuating responses (around the value observed for 0 μM) are noted for both treatments (PER and GP114) up to 10 mM and statistically significant differences (between control and respective experimental group) may be observed from 10 μM. Curiously, although a decrease in survival can be observed at 10 and 20 mM for PER, it is not seen at 40 mM, possibly due to compensation by other members of this gene family at higher concentrations (Figure 2A). On the other hand, GP114 also shows a decrease between experimental group and respective control at 20 mM, which is still present at 40 mM (Figure 2B), suggesting that a similar compensatory mechanism is not present, or is less effective. Taken together, these findings suggest that both PER and GP114 are involved in controlling oxidative stress, a situation known to occur during dehydration.

### Evaluation of soaking as a means of triggering RNAi in *P. superbus*

**Figure 3:**
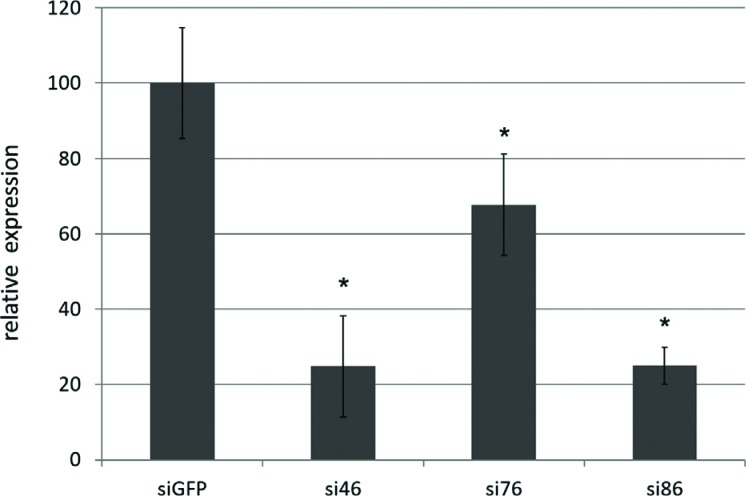
Molecular confirmation of gene knockdown by semi-quantitative RT-PCR. Three targets were selected to confirm gene silencing by soaking with dicer substrates, two of which lead to a decrease in survival after desiccation (si46 and si76) and one of which did not (si86). * p<0.05 (Tukey, compared to control group).

We decided to determine whether immersing *P. superbus* in solutions containing long (>100 bp) and short (27 bp) RNA duplexes was also effective in promoting knockdown. We tested a few representative targets to show that, as judged by semi-quantitative RT-PCR, successful gene silencing was achieved by soaking with 27 bp RNA duplexes (known as dicer substrates) at 1 μM for 24 h (Figure 3 and Figure S2). Soaking in solutions of RNA duplexes resulted in gene silencing in a dose-dependent manner (up to 1 μM), although nonspecific effects began to emerge at high concentrations (10 μM, data not shown). Successful RNAi using dicer substrates was confirmed by its effect on target mRNA levels and by phenotypical analyses (knockdown of *ifb-1* and *actin* genes, data not shown).

### Functional identification of anhydrobiosis-related genes in *P. superbus*

**Figure 4:**
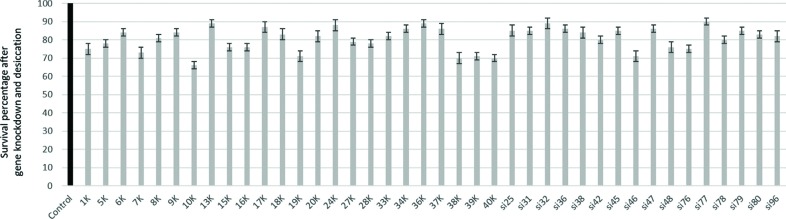
Identification of anhydrobiosis-related genes in *P. superbus*. All the 40 genes here indicated lead to statistically significant reductions >10% in survival percentage (z-test, p<0.05 compared to control group) after knockdown and desiccation.

We combined the use of the staining method for assessment of nematode viability with the soaking method for induction of RNAi in a medium-scale screening experiment to identify *P. superbus* genes associated with anhydrobiosis. We selected a panel of 97 genes, from which 40 genes (Figure 4 and Table 1) showed reduced survival (20% on average) after knockdown and desiccation challenge. This level of decrease has been previously observed in other nematodes (Reardon *et al.*, 2010; Erkut *et al.*, 2013) and revealed to be consistent with involvement in anhydrobiosis.

Approximately half of the positive targets (24 genes) were related to cell signalling (kinase domain-containing proteins). The remaining sixteen genes encode several classes of proteins, including proteases, ribosomal proteins, structural proteins, aquaporins, DNA repair enzymes and molecular chaperones.

## Discussion

*P. superbus* is a well-studied anhydrobiotic nematode that is amenable to RNAi (Shannon *et al.*, 2008) and therefore a gene silencing approach can be taken to test the involvement of candidate genes in anhydrobiosis. However, to achieve this on anything more than a small scale requires some improvement to screening procedures, particularly in assessing survival of desiccation. Prior to the work in this report, desiccation tolerance was determined by observation under the microscope of “movement” as a survival criterion. However, this has its drawbacks, since absence of mobility is not necessarily evidence of death, and therefore the surviving fraction may be underestimated; it is also time- and labor-intensive. We describe here an alternative staining method, which is simple, fast, cheap and provides unequivocal survival data; it is particularly suited to medium- or large-scale screening experiments. Of the two stains tested, erythrosin B may be slightly preferable, as it has been used as a food colorant for many years and therefore carries fewer safety concerns.

The developed approach could be used to deepen our understanding on the functional roles of two *P. superbus* genes, involved in oxidative stress, in the process of desiccation tolerance. GP114 has previously been validated as an anhydrobiosis-related gene using RNAi (Reardon *et al.*, 2010); we confirmed this here and also showed the involvement of PER, demonstrating a role for peroxiredoxins in anhydrobiosis for the first time. As their name suggests, peroxiredoxins reduce hydrogen peroxide levels and thus help to limit oxidative damage caused by water loss, including lipid peroxidation, protein oxidation and DNA mutations, which may otherwise compromise cell function, eventually culminating in cell death (Hansen *et al.*, 2006). The decrease in viability (~60%, compared to control group) after desiccation of PER-silenced nematodes reveals that peroxiredoxin activity is important for successful anhydrobiosis in *P. superbus*. However, the complexity of this phenomenon means that many other processes must be involved, and many studies have highlighted the importance of non-reducing disaccharide accumulation, LEA proteins, heat shock proteins and other molecular adaptations (see Erkut *et al.*, 2013 for a systems approach in *C. elegans* and Burnell and Tunnacliffe (2011) for an earlier review of nematode anhydrobiosis).

Once we had validated the staining protocol by showing the involvement of GP114 and PER in *P. superbus* anhydrobiosis, our next step was to develop a practical approach to screen a larger set of genes. Therefore, we decided to evaluate the efficiency of RNAi by soaking in this species, since it is faster than feeding methods (24 h, instead of several days on feeding plates), uses less space (0.2 - 1.5 mL tubes, instead of 60 - 90 mm plates), demands fewer consumables and does not require cloning cDNAs in special feeding vectors. Original studies in *C. elegans* showed that SID-1, a transmembrane protein expressed in the pharynx, is responsible for the uptake of long dsRNA (Feinberg and Hunter, 2003) and short siRNAs at high concentrations (Issa *et al.*, 2005; Shih *et al.*, 2009). The observation of nonspecific effects at extremely high concentrations of dicer substrates (10 μM) is probably due to: (i) interference in endogenous pathways, such as microRNA biosynthesis (Grimm *et al.*, 2006), (ii) off-targeting, *i.e.*, silencing other genes, or/and (iii) general interference in the transcriptome (Jackson *et al.*, 2003).

We then initiated a medium-scale screening experiment in *P. superbus* with 97 genes implicated in anhydrobiosis using RNAi by soaking and observed a decrease in survival for nearly half of them. Many of these gene sequences, together with their possible roles in anhydrobiosis, were first discussed by Tyson *et al.* (2012) who identified them in *P. superbus* after generating an EST library. Since this library was constructed using mixed populations of worms (no specific developmental stages) under normal humidity conditions, this study was not able to determine whether the genes identified were in fact involved in anhydrobiosis. Here we will discuss some of the genes which were not explicitly mentioned in previous work (Tyson *et al.*, 2012) and which our RNAi experiments suggest to have a functional role in *P. superbus* anhydrobiosis.

The most abundant EST found in *P. superbus* (Tyson *et al.*, 2012) encodes sxp/Ral-2 protein, a small (16–21 kDa) basic protein with a common domain of unknown function, which is highly expressed in parasitic nematodes and is secreted onto the surface of the worm cuticle. Several cuticle proteins are differentially expressed during desiccation in diverse species (Adhikari *et al.*, 2009; Cornette *et al.*, 2010), which are possibly involved in modification of cuticle permeability and which, along with aquaporins, surface lipids (Wharton *et al.*, 2008) and behavioral responses (worm coiling/clumping), might promote controlled water loss during dehydration. We were able to validate an anatomically related polypeptide: the `cuticle collagen protein LON-3’ (target code “si31”), a polypeptide involved in *C. elegans* body shape and probably targeted by TGF-beta signaling (Suzuki *et al.*, 2002). Notably, the surface of *C. elegans* displays indentations in its circumference spaced about one micrometer apart, defining rings called annuli. It is suggested that annuli may function as pleats, allowing the cuticle to fold on the inner radius of a bend and extend over the outer radius (Riddle *et al.*, 1997). It was recently shown that *C. elegans lon-2* mutants present with wider annuli and a decrease in furrow depth (Essmann *et al.*, 2017). Therefore, morphological changes promoted by *lon-3* (which is closely related to *lon-2*) possibly involve alterations in annuli also, thereby altering the total body surface and controlling water loss.

Schokraie *et al.* (2010) found three different cathepsins (K, Z and L1) during a proteomic study in the tardigrade *M. tardigradum*. One of them, cathepsin-L-like cysteine proteinase (target code “si25”), which is a ubiquitous protease in eukaryotes, is associated with desiccation tolerance in *P. superbus*. The parasitic nematode *Parelaphostrongylus tenuis* expresses a cathepsin B cysteine protease homolog which is abundant in larval stages. Although it is less abundant in larval stage L3, it is still predominant during this developmental phase. Curiously, L3 is the phase when the parasite leaves the intermediate host (snail) and it was observed that, to some extent, L3 is desiccation tolerant, allowing persistence in the environment (Duffy *et al.*, 2006). Although most cathepsins degrade autophagosomal content, cathepsin L also degrades lysosomal membrane components (Kaminskyy and Zhivotovsky, 2012). Therefore, this enzyme could be involved in a general turnover of damaged proteins after desiccation. Several other proteases have been implicated in desiccation tolerance in diverse species, including (i) serine endopeptidases and aminopeptidases in the resurrection plant *Ramonda serbica* (Kidric *et al.*, 2014), (ii) ATP-dependent ClpXP protease in the bacterium *Staphylococcus aureus* (Chaibenjawong and Foster, 2011), and (iii) carboxy-terminal protease (CtpA) in *Rhizobium leguminosarum* (Gilbert *et al.*, 2007), indicating an important role in proteome turnover mediated by proteinases.

Since *P. superbus* is a fast desiccation strategist, *i.e.* it may enter anhydrobiosis without preconditioning, it is likely that a substantial proportion of its proteome is constitutively primed to enter the dry state. Therefore, the relatively large number of kinases (24) revealed by our RNAi experiments to be important for anhydrobiosis may act as essential signaling regulators or as activators of the proteome, by phosphorylating their substrates within a very short period, allowing rapid entry in the dry state. Other studies have also demonstrated a high number of such proteins involved in dehydration/drought/desiccation tolerance in different plant species, including 229 kinases in chrysanthemum plants (Xu *et al.*, 2013) and over 460 kinases in the resurrection plant *Myrothamnus flabellifoliathe* (Ma *et al.*, 2015). Still within this context, adenylate kinase (target code “19K”), an enzyme involved in the biosynthesis of ATP, was demonstrated to be up-regulated in a drought-tolerant genotype of tomato (Gong *et al.*, 2010). In *P. superbus*, accumulation of ATP during dehydration is probably a key aspect of the whole process, providing a readily available source of energy during rehydration, when cells need energy to resume activities, but are not fully capable of generating it.

An important aspect of our approach relies on the fact that since our panel of 97 targets were genes differentially expressed during desiccation in different anhydrobiotic species (the nematode *Plectus murrayi*, the dipteran *Polypedilum vanderplanki* and two tardigrades *Hypsibius dujardini* and *Milnesium tardigradum*), one might expect that most (if not all) of them would be shown to be anhydrobiosis-related in *P. superbus*. However, our study confirmed the association of only 40 of them. This result may reflect that different anhydrobiotic species adopt different biochemical strategies and/or molecular programs to promote desiccation tolerance. For example, although many anhydrobiotic animals accumulate trehalose during desiccation, bdelloid rotifers do not (Lapinski and Tunnacliffe, 2003), while plants often accumulate sucrose (Zhang *et al.*, 2016). Moreover, *P. superbus* is a fast strategist (*i.e.*, it is able to enter anhydrobiosis in the absence of preconditioning), while other species demand a slow desiccation protocol (Shannon *et al.*, 2005). Thus, we cannot unequivocally rule out the involvement of the other 57 assessed targets in anhydrobiosis (Table 1, in white) since their roles may be minor (secondary) within anhydrobiosis, or be compensated by other genes, demanding other genetic analyses (*e.g.*, CRISPR-mediated single- or multiple-knockouts, which is not established for this species) to determine it.

The set of genes identified here, along with other similar functional studies, might be used for the development of anhydrobiotic engineering (García De Castro *et al.*, 2000), a research field which aims to render cells and whole organisms tolerant to dehydration. Several anhydrobiosis-based approaches have recently been developed in order to preserve biological samples at room temperature for longer periods, including the stabilization of RNA molecules (Hernandez *et al.*, 2009) and poxviral/adenoviral vaccines without refrigeration (Alcock *et al.*, 2010). Further challenges encompass the development of transgenic plants tolerant to extreme drought, a recurrent and increasing challenge in agriculture and food production. This may be even more relevant considering the expected population growth over the next decades and the predictions of a rise in global temperature. Heterologous expression of just one desiccation-related protein is sufficient to promote a significant increase in drought tolerance (Wang *et al.*, 2017). For example, Liu *et al.* (2009) produced transgenic tobacco plants expressing LEA proteins derived from the resurrection plant *Boea hygrometrica.* Transgenic plants expressing BhLEA1 (*Boea hygrometrica* LEA1) or BhLEA2 were submitted to water stress and, compared to a control group, displayed (i) higher water content, (ii) higher activities of photosystem II, superoxide dismutase and peroxidase, (iii) lower membrane permeability and (iv) stabilization of several proteins including ribulose-bisphosphate carboxylase (large subunit).

On the other hand, medicine might also benefit from discoveries on the molecular basis of anhydrobiosis. This might be achieved via strategies based on the concept of ‘DNA vaccines’, which promote the transient expression of heterologous proteins within the human body. This approach allows, for example, the expression of virus-derived proteins in the body, which, in turn, trigger the immune system to produce corresponding antibodies and promote protection (Dowd *et al.*, 2016). Such an approach might be used to express anhydrobiosis-related genes for novel purposes. For example, Hashimoto *et al.* (2016) reported several unique genes in the anhydrobiotic tardigrate *Ramazzottius varieornatus*. One of these was shown to suppress X-ray-induced DNA damage by nearly 40% and to improve radiotolerance when expressed in human cell cultures. Therefore, heterologous expression of the “DNA repair protein RAD51 homolog 1” (target si42, identified in our present study) via strategies based on the concept of `DNA vaccines’ might eventually be useful for people submitted to radiotherapy during treatment against cancer. More elaborate and complex strategies might allow, in the future, the expression of several anhydrobiosis-related proteins and the preservation in the dry state and at room temperature of human organs for transplant until a compatible patient is found.

Finally, the current work, along with the impending publication of the *P. superbus* genome by other groups, helps to establish *P. superbus* as a nematode model for anhydrobiosis. Comparative studies between *P. superbus* and *C. elegans*, which have a fundamentally different approach to desiccation tolerance, may shed light on the evolution of desiccation tolerance in these proximal species.

## Conclusions

We have found evidence for the participation of 40 anhydrobiosis-related genes in *P. superbus*. Our data, along with transcriptomic and proteomic analyses, help unveil the genetic scaffold underlying extreme desiccation tolerance, as well as the development of anhydrobiotic engineering.

## Supplementary material

The following online material is available for this article:

Table S1 - Nucleotide sequences of primers.

Table S2 - Nucleotide sequences of all dicer substrates.

Figure S1 - Staining *P. superbus* with erythrosin B and trypan blue.
