# Supplementary material for: Multiple genes contribute to anhydrobiosis (tolerance to extreme desiccation) in the nematode Panagrolaimus superbus
Source: Genet Mol Biol. 2017 Nov 6;40(4):790–802. doi: 10.1590/1678-4685-GMB-2017-0030 (PMC5738622; doi:10.1590/1678-4685-GMB-2017-0030)
Supplement: Supplementary file 1 [file 1415-4757-gmb-1678-4685-GMB-2017-0030-Suppl01.pdf]

Supplementary Material to “Multiple genes contribute to anhydrobiosis (tolerance to extreme desiccation) in the nematode Panagrolaimus superbus”

Table S1 - Nucleotide sequences of primers. pDNR-LibT7 primers were used to amplify kinase genes cloned in the pDNR-library. These amplicons contained T7 promoters at each end. Primers: actin, PER, GP114, si46, si76 and si86, were used for knockdown confirmation via RT-PCR. Primers 1 to 21 were used to clone the corresponding *P. superbus* sequences (see Table 1) via RT-PCR. Primers “All T7::primers from 1 – 21” were used to produce the corresponding amplicons with T7 promoters at each end.

Targets	strand	sequence (5’-3’)
pDNR-LibT7	forward	GGTAATACGACTCACTATAGATACATTATACGAAGTTATCA
	reverse	GGTAATACGACTCACTATAGATGACCATGTTCACTTACCTA
actin	forward	GCCGGGCTTGATGAAAATTCT
	reverse	TGTCGGCAATACCAGGATACA
PER	forward	CACATCCTGCACATTTTACGC
	reverse	TGCAACGAATCAAGGACTCTG
GP114	forward	CGGCATGTTTGATTGTGGCAT
	reverse	TTTGTGGGCAGCTATCCTTT
si46	forward	AAACTTCTGCTGAATCGTGGG
	reverse	AGAAACAGTTGAGACACCAGG
si76	forward	GCAACTTGATACAGCTCTTCG
	reverse	TTGAACTGCTGTTCTACCTGG
si86	forward	GGGGCCAATTGTATCAAGATC
	reverse	AAAAAAATCACATAAAAGAGTCAGA
1	forward	GGTTTTAGAGGGTGCCATGTA
	reverse	AGTTTATTGATTTCCCAAAAGCC
2	forward	AGTTTTGTCAATACTGATTCTCC
	reverse	GCAGGTGTTGACCACTCATTT
3	forward	GGGTGTTGGAGATATGTTCGT
	reverse	ATATAAGTCAACAACAAAAACGATT
4	forward	GGGTACTAAAGATGGGACAGA
	reverse	AAGAAGAGTTTTTCCAGTGCCA
6	forward	GGGGAATTAATTGATCGAGGG
	reverse	GATTTAAGCATGCTGGGATCAA
7	forward	ACTAAAATGATTAAAGCTGGAACT
	reverse	ATGTTGCAGATGAAAGAGGTAC
8	forward	GGTGCTTGGAATCCTGTTGAT
	reverse	AAAGGAGATTAAACAGCCATATC
9	forward	GTCTTTGCTGGTGTTGGTGAA
	reverse	GAGAAACGAATTTGCCTTGATG
10	forward	GGGGATCATTATTGACTTTTTATT
	reverse	AGATACAAAGAAACCCCGATAAA
11	forward	TGTCTTCGACGAGATATTTCTG
	reverse	TTTGCGACTAATTCATTTATTCATA
15	forward	GTTAAATCAGCATCGAGGAATC
	reverse	GAATGAATTTATGAACGATGGCT
16	forward	AAGATGGCACGTCGTTATGAC
	reverse	CAGAAGAAGTTTCGCATCAAGA
17	forward	GGGGAATTCAGTATTTTACCTC
	Reverse	GAGTTCTGGAACATTCTTCACT
21	Forward	TTATTTCGTTTGTGTACTTTCTTC
	Reverse	CAGAGAGAAATTACTTGGCTTC
All T7::primers from 1 - 21	Forward	Add at 5’: GGTAATACGACTCACTATAG-primer sequence
	Reverse	Add at 5’: GGTAATACGACTCACTATAG-primer sequence
