# Supplementary material for: Multiple genes contribute to anhydrobiosis (tolerance to extreme desiccation) in the nematode Panagrolaimus superbus
Source: Genet Mol Biol. 2017 Nov 6;40(4):790–802. doi: 10.1590/1678-4685-GMB-2017-0030 (PMC5738622; doi:10.1590/1678-4685-GMB-2017-0030)
Supplement: Supplementary file 2 [file 1415-4757-gmb-1678-4685-GMB-2017-0030-Suppl02.pdf]

# Supplementary Material to “Multiple genes contribute to anhydrobiosis (tolerance to extreme desiccation) in the nematode Panagrolaimus superbus”

**Table S2** - Nucleotide sequences of all dicer substrates.

| code | target | Strand | Sequence (5’-3’) |
|---|---|---|---|
| GFP | Green Fluorescent Protein (control) | sense | GCGUUCAAUUAGCAGACCAUUAUCAAC |
| | | antisense | UGAUAAUGGUCUGCUAAUUGAACGCUU |
| si23 | Pinin/SDK/memA/ protein conserved region containing protein | sense | GGUGCACGAGAUCGCCUAAUGUUUGGU |
| | | antisense | CAAACAUUAGGCGAUCUCGUGCACCAU |
| si24 | glutamate dehydrogenase | sense | ACUUCAAACUGUAUCUACUUUAAUGAC |
| | | antisense | CAUUAAAGUAGAUACAGUUUGAAGUUC |
| si25 | cathepsin L-like cysteine proteinase | sense | GGAUCUCUUGAAGGACAACAUAAACGU |
| | | antisense | GUUUAUGUUGUCCUUCAAGAGAUCCGG |
| si26 | A - heat shock protein 70 | sense | UUCGAUCAUUUUUUACAUUUAAUAUUU |
| | | antisense | AUAUUAAAUGUAAAAAAUGAUCGAAUG |
| si27/28 | B/C - Heat shock 70 kDa protein | sense | CUGUCCAGGACUUGCUUCUUCUUGAUG |
| | | antisense | UCAAGAAGAAGCAAGUCCUGGACAGUU |
| si29 | Ras-related protein Rab-1A | sense | CUGGUGGCUGCUGCUAAAUUAUCAAUU |
| | | antisense | UUGAUAAUUUAGCAGCAGCCACCAGUU |
| si30 | Ras-related protein Rab-11B | sense | GGUGCCCUUCUUGUCUACGAUAUUGGU |
| | | antisense | CAAUAUCGUAGACAAGAAGGGCACCAA |
| si31 | cuticle collagen protein LON-3 | sense | AGAUGGGCAAGGUGCUGAAGAUAUUAC |
| | | antisense | AAUAUCUUCAGCACCUUGCCCAUCUUU |
| si32 | CBR-RPS-0 protein (40S ribosomal protein AS) | sense | UGGGCUGCUUCAUCUUGGGAUAAUACU |
| | | antisense | UAUUAUCCCAAGAUGAAGCAGCCCAAU |
| si33 | Immunodominant antigen Ov33-3 / Pepsin inhibitor Dit33 | sense | AACAACAAGUUAAAGAAUUAUUUGGUG |
| | | antisense | CCAAAUAAUUCUUUAACUUGUUGUUGA |
| si34 | Ubiquitin-conjugating enzyme H1 | sense | GUUGGGCUGCUUCUCUUUCUCUUCGUA |
| | | antisense | CGAAGAGAAAGAGAAGCAGCCCAACGA |
| si35 | histone H2B 2 | sense | UGCUGCUGUUGAUGAUGUUGUAAAUGU |
| | | antisense | AUUUACAACAUCAUCAACAGCAGCAAA |
| si36 | cytochrome P450 like_TBP | sense | UCAGAGCACUGGGCAGAAAUCACAAAU |
| | | antisense | UUGUGAUUUCUGCCCAGUGCUCUGAAU |
| si37 | CRE-RPL-9 protein | sense | CGUCAUGCUUCUAUGGAUUUAACCCGU |
| | | antisense | GGGUUAAAUCCAUAGAAGCAUGACGAA |
| si38 | ribosomal protein L44 | sense | ACAGGGCUCAUCAUGGUUAAUGUACUU |
| | | antisense | GUACAUUAACCAUGAUGAGCCCUGUUU |
| si39 | A - euk. Transl. Elong. factor 1A | sense | UCUGGAUUCAAUGGCGAUAACAUGCUU |
| | | antisense | GCAUGUUAUCGCCAUUGAAUCCAGAGA |
| si40 | B - euk. Transl. Elong. factor 1A | sense | UAUUGCUCUCUGGAAAUUUGAAACUGC |
| | | antisense | AGUUUCAAAUUUCCAGAGAGCAAUAUC |
| si41 | Elongation factor 1 beta | sense | CUGCUGGAGGUGAUGAUGACUUUGAUU |
| | | antisense | UCAAAGUCAUCAUCACCUCCAGCAGCU |
| si42 | DNA repair protein RAD51 homolog 1 (4e-110) | sense | ACUGGGAGCUUGACUGAAAUAUUUGGU |
| | | antisense | CAAAUAUUUCAGUCAAGCUCCCAGUUU |
| si43 | Pv-hsp60 | sense | AUGGGUGGUGGCAUGUUCUAAAAUGUG |
| | | antisense | CAUUUUAGAACAUGCCACCACCCAUAC |
| si44 | Pv-p23 | sense | AUUGGUUGAAAAAGUUUAGUUUAAGAU |
| | | antisense | CUUAAACUAAACUUUUUCAACCAAUUU |
| si45 | putative heat shock protein 90 | sense | UUGUCCUCUCUUGCUGAUUAUGUUUGG |
| | | antisense | AAACAUAAUCAGCAAGAGAGGACAACU |
| si46 | 60S ribosomal protein L4 | sense | UGGUCGUGCCGUAGCUCGUAUUCCUCG |
| | | antisense | AGGAAUACGAGCUACGGCACGACCAGU |
| si47 | 40S ribosomal protein S8 | sense | AAGGGUGCUAAGCUUAAUGAAGAAGAA |
| | | antisense | CUUCUUCAUUAAGCUUAGCACCCUUCU |
| si48 | 60S ribosomal protein L7a | sense | AUCGUCCAGAAACCAAACAACAAAAAU |
| | | antisense | UUUUGUUGUUUGGUUUCUGGACGAUAU |
| si76 | oxidoreductase, aldo/keto reductase family protein | sense | AACAGAUUACAUUGAUUUAUAUUUAAU |
| | | antisense | UAAAUAUAAAUCAAUGUAAUCUGUUCG |
| si77 | | sense | UCCGCUGCUCUGUCUCUUUCUCUUCCA |

| code | target | Strand | Sequence (5'-3') |
|---|---|---|---|
| | zinc finger domain containing protein (AN1-like Zinc finger) | antisense | GAAGAGAAAGAGACAGAGCAGCGGAUG |
| si78 | channel protein, MIP family; aquaporin | sense | CUGCUGUCACCCUUGCCUUCCUUGUUC |
| | | antisense | ACAAGGAAGGCAAGGGUGACAGCAGGA |
| si79 | autophagy-related protein 2-like protein A | sense | GUACUUGCCUUAUUGUUUUGUAUUUUG |
| | | antisense | AAAUACAAAACAAUAAGGCAAGUACAA |
| si80 | peptidyl-prolyl cis-trans isomerase domain containing protein; cyclophilin-type peptidyl-prolyl cis-trans isomerase-15 | sense | AGGCUCCAGUCGAAUAAGUGCUUUACC |
| | | antisense | UAAAGCACUUAUUCGACUGGAGCCUUU |
| si81 | chaperonin Cpn60 TCP-1 domain containing protein | sense | CUUCCCGGUGGUACUGUUAAAGAUUCU |
| | | antisense | AAUCUUUAACAGUACCACCGGGAAGCU |
| si82 | Derlin-2 | sense | UUGCUUGCGGUCAUUUGUAUUAUUUCU |
| | | antisense | AAAUAAUACAAAUGACCGCAAGCAAUU |
| si83 | DJ-1 | sense | GACCCUUGCGAGACGAACUACAGCGAA |
| | | antisense | CGCUGUAGUUCGUCUCGCAAGGGUCCA |
| si84 | Ezrin Radixin Moesin family member (erm-1) | sense | CAAGCCCGUGUCCAAGAUGGUCUUAGU |
| | | antisense | UAAGACCAUCUUGGACACGGGCUUGAU |
| si85 | HSP70 cochaperone BAG1 | sense | ACAUGACUGCUGAAAUGAUUACACGAU |
| | | antisense | CGUGUAAUCAUUUCAGCAGUCAUGUCG |
| si86 | LC3, GABARAP and GATE-16 family member (lgg-1) | sense | UGGACUCAACUCAGCAAAACGAUAACU |
| | | antisense | UUAUCGUUUUGCUGAGUUGAGUCCAAU |
| si87 | ATP-dependent protease La; lon protease homolog, mitochondrial precursor | sense | UUGCAAUGACUGGUGAAAUUUCUUUAA |
| | | antisense | AAAGAAAUUUCACCAGUCAUUGCAACA |
| si88 | isocitrate dehydrogenase, NADP-dependent | sense | GGCCGGGGCAUCUGGUAUGAACAUCGU |
| | | antisense | GAUGUUCAUACCAGAUGCCCCGGCCGU |
| si89 | prefoldin subunit 2, PFD-2 | sense | CUACCGACUUAGUGAUAUCUUAGUAGA |
| | | antisense | UACUAAGAUAUCACUAAGUCGGUAGAA |
| si90 | Probable E3 ubiquitin-protein ligase | sense | GUGUCAGAGAACGGUAAAUCAAUGAAA |
| | | antisense | UCAUUGAUUUACCGUUCUCUGACACCA |
| si91 | Proteasome subunit alpha type 4 | sense | ACGCGGCACGAUAAUAAGACUGUCAUC |
| | | antisense | UGACAGUCUUAUUAUCGUGCCGCGUAA |
| si92 | CRE-PBS-1 protein; proteasome domain containing protein | sense | UCGGGAUCAAAUGAAUGCAAGUGUUAU |
| | | antisense | AACACUUGCAUUCAUUUGAUCCCGAUA |
| si93 | Protein disulfide isomerase | sense | AUAGUCGCAAAUUAAAAUACAGUACCA |
| | | antisense | GUACUGUAUUUUAAUUUGCGACUAUUU |
| si94 | RIC1 Putative stress responsive protein | sense | UGAGUAUUGUAUUAAUGUUUAAAUAAA |
| | | antisense | UAUUUAAACAUUAAUACAAUACUCAAA |
| si95 | Small heat shock proteinalpha crystallin family | sense | UUUCUUCUCAUCGCAUAUAAUAUUUUU |
| | | antisense | AAAUAUUAUAUGCGAUGAGAAGAAAGU |
| si96 | tetratricopeptide TPR-1 domain containing protein; hsp70-interacting protein, putative | sense | UGGCGGGUGGAAACAAUUCUGAAACAA |
| | | antisense | GUUUCAGAAUUGUUUCCACCCGCCAUU |
| si97 | THaumatiN family member | sense | GGGCUCGAACAGGGUGUGAUGACUCUU |
| | | antisense | GAGUCAUCACACCCUGUUCGAGCCCAA |
| si98 | Ubiquitin conjugating enzyme (E2) family member (ubc-3) | sense | AUGGAUGCUGAUGAAGAUUAUUAUGAU |
| | | antisense | CAUAAUAAUCUUCAUCAGCAUCCAUAA |
| si99 | ubiquitin | sense | UGGCCGUACACUCUCUGAUUAUAACAU |
| | | antisense | GUUAUAAUCAGAGAGUGUACGGCCAUC |
| si100 | ubiquitin-activating enzyme E1 | sense | AUCUUCGUGCUGCAAAUUAUGAUAUCA |
| | | antisense | AUAUCAUAAUUUGCAGCACGAAGAUUU |
