# Supplementary material for: Multiple genes contribute to anhydrobiosis (tolerance to extreme desiccation) in the nematode Panagrolaimus superbus
Source: Genet Mol Biol. 2017 Nov 6;40(4):790–802. doi: 10.1590/1678-4685-GMB-2017-0030 (PMC5738622; doi:10.1590/1678-4685-GMB-2017-0030)

Supplementary Material to "Multiple genes contribute to anhydrobiosis (tolerance to extreme desiccation) in the nematode Panagrolaimus superbus"

Figure S1 - Staining *P. superbus* with erythrosin B and trypan blue. A) Live *P. superbus* (negative control) are not stained with erythrosin B. A very small percentage (<3%) of live adults displayed small, lightly stained dots. B) Heat-killed *P. superbus* (positive control) worms are stained with erythrosin B. C) Mixed population of *P. superbus* (heat-killed and living) after soaking in erythrosin B. D) Live *P. superbus* (negative control) are not stained with trypan blue. E) Heat-killed *P. superbus* (positive control) worms are stained with trypan blue. F) Mixed population of *P. superbus* (heat-killed and living) after soaking in trypan blue.

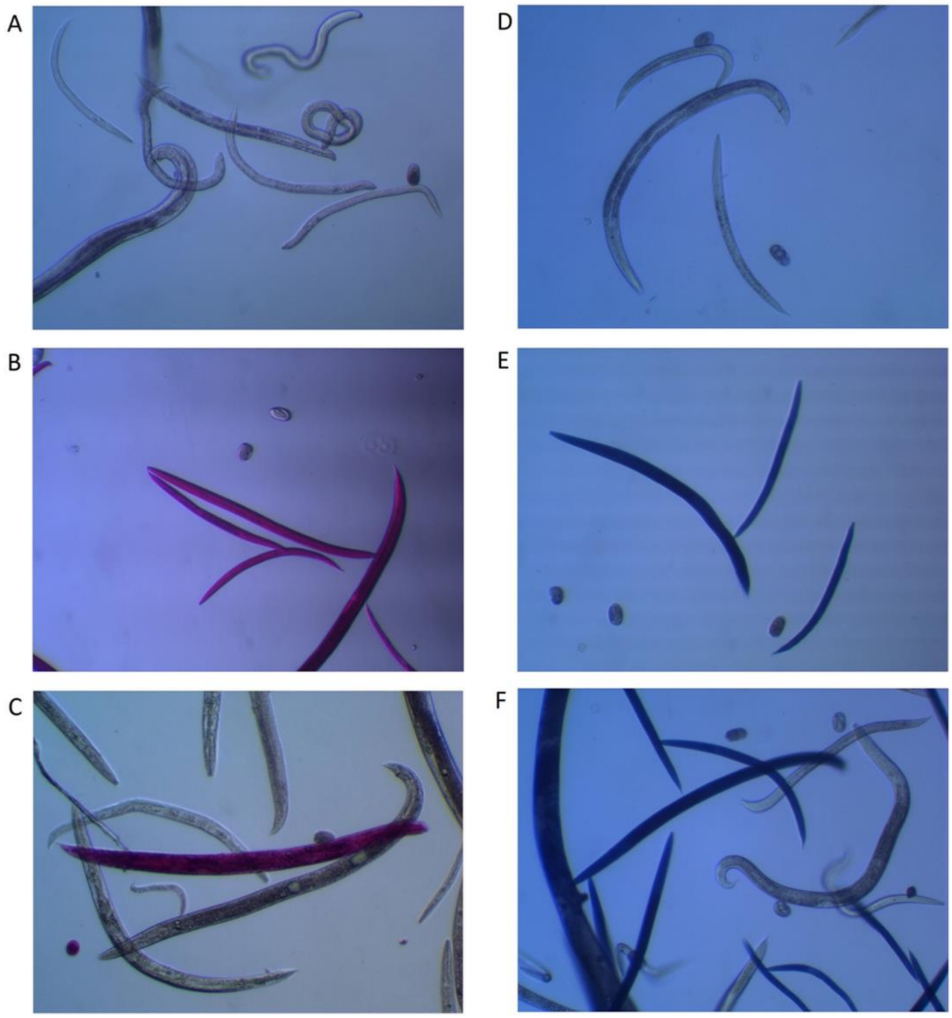

Supplement: Supplementary file 3 [file 1415-4757-gmb-1678-4685-GMB-2017-0030-Suppl03.pdf]
