# Supplementary material for: Multiple genes contribute to anhydrobiosis (tolerance to extreme desiccation) in the nematode Panagrolaimus superbus
Source: Genet Mol Biol. 2017 Nov 6;40(4):790–802. doi: 10.1590/1678-4685-GMB-2017-0030 (PMC5738622; doi:10.1590/1678-4685-GMB-2017-0030)

Supplementary Material to "Multiple genes contribute to anhydrobiosis (tolerance to extreme desiccation) in the nematode Panagrolaimus superbus"

Figure S2 - Gel densitometry. Worms were soaked in solutions containing 1 μM of siRNA against GFP (siGFP, the control group) or against the *ifb-1* gene (si-ifb1). Left: total RNA was extracted and RT-PCR was performed to amplify target gene (*ifb-1*, the thinner band) and the endogenous control (*β-actin*). Right: gel densitometry using the software ImageJ.

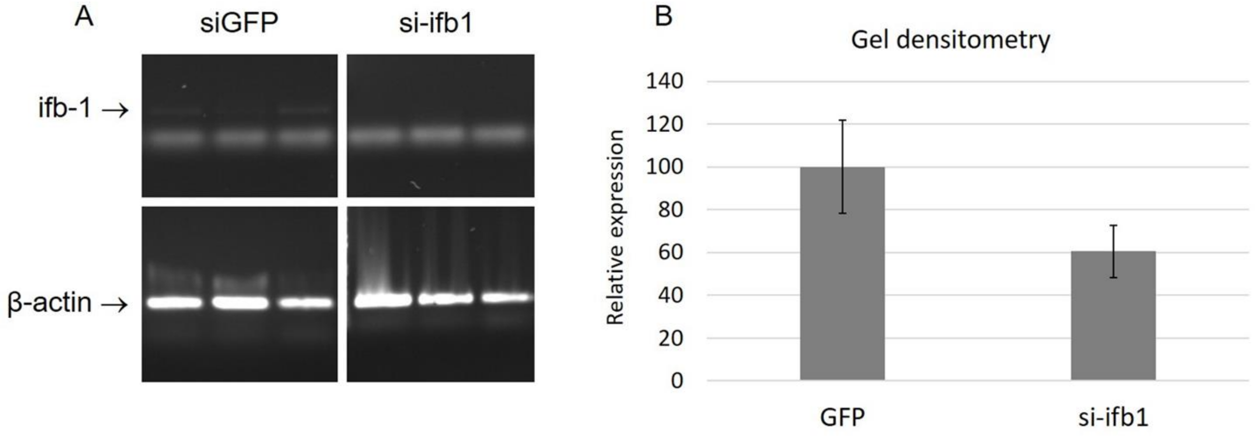

Supplement: Supplementary file 4 [file 1415-4757-gmb-1678-4685-GMB-2017-0030-Suppl04.pdf]
